# Sunshine and Sadness: A Case Report on Summer Season Depression

**DOI:** 10.7759/cureus.75190

**Published:** 2024-12-05

**Authors:** Nikita Shidhore, Ajish Mangot

**Affiliations:** 1 Psychiatry, Krishna Institute of Medical Sciences, Krishna Vishwa Vidyapeeth (Deemed to be University), Karad, IND

**Keywords:** depression, india, seasonal affective disorder, spaq, summer season, tropical

## Abstract

Seasonal affective disorder (SAD) is typically associated with winter; however, its less common variant, summertime depression, presents with depressive episodes during the summer months. We report a case of a 46-year-old male patient with recurrent summertime depressive episodes characterized by low mood, fatigue, anhedonia, insomnia, and loss of appetite, each resolving with the onset of the winter season. Our patient’s history of summertime depression aligned with the atypical SAD symptoms, including irritability and weight loss, commonly associated with non-seasonal depression. A diagnosis of major depressive disorder, moderate severity, with a seasonal pattern was confirmed using the Diagnostic and Statistical Manual of Mental Disorders, Fifth Edition, Text Revision criteria, and the Seasonal Pattern Assessment Questionnaire. The patient was initiated on desvenlafaxine 50 mg once a day with a dose titrated to 100 mg once a day in two weeks, while his previous mood stabilizer oxcarbazepine 600 mg in two divided doses was continued. He achieved remission within four to six weeks with his Hamilton Rating Scale for Depression score decreasing from 18 to seven. Our case underscores the importance of recognizing seasonal patterns in affective disorders within tropical climates, like India, and highlights potential environmental and physiological mechanisms, such as heat stress and immune responses, contributing to summertime SAD.

## Introduction

Seasonal affective disorder (SAD) is primarily characterized by recurrent episodes of depression that correlate with seasonal changes, most commonly occurring in winter [1]. However, a less common variant, often referred to as summertime depression, presents with depressive symptoms during the summer months. Historically, SAD was first noted in the 19th century, with clinicians documenting the relationship between light exposure and depressive symptoms [2]. The formal recognition of SAD as a distinct clinical entity emerged in the 1980s, with significant contributions from Rosenthal and colleagues [3].

According to the Diagnostic and Statistical Manual of Mental Disorders, Fifth Edition, Text Revision (DSM-5-TR), SAD is recognized as a specifier for recurrent major depressive disorder [4]. The diagnostic criteria are summarized as follows: (A) A consistent temporal association exists between episodes of major depression and a specific time of the year. (B) These depressive episodes are followed by full remission during a characteristic seasonal period. (C) Over the past two years, at least two major depressive episodes must demonstrate this seasonal pattern without any episodes occurring outside the specified season. (D) The frequency of seasonally patterned depressive episodes must significantly exceed that of non-seasonal depressive episodes.

Epidemiological studies suggest that the prevalence of SAD varies geographically, with estimates ranging from 1% to 9% in temperate regions [5]. While winter-onset SAD is more frequently reported, summertime depression has garnered attention in clinical literature, though it is recognized as a rarer phenomenon [6]. In India, awareness of both SAD and summertime depression is limited, with a paucity of studies addressing this condition [6]. Thus, although the preliminary evidence suggests that affective disorders related to seasonal fluctuations do occur, particularly in regions experiencing extreme temperature variations, the prevalence of summertime depression specifically remains underresearched.

This case report describes a middle-aged male patient who presented to the psychiatry outpatient department with symptoms of a depressive episode in the month of July. Detailed history-taking revealed that the patient had experienced similar depressive episodes in previous years, each coinciding with the onset of summer. Our report highlights the relatively underexplored phenomenon of summertime depression, aiming to elucidate its clinical presentation, possible underlying mechanisms, and broader implications for future research and clinical practice.

## Case presentation

**Figure 1:**
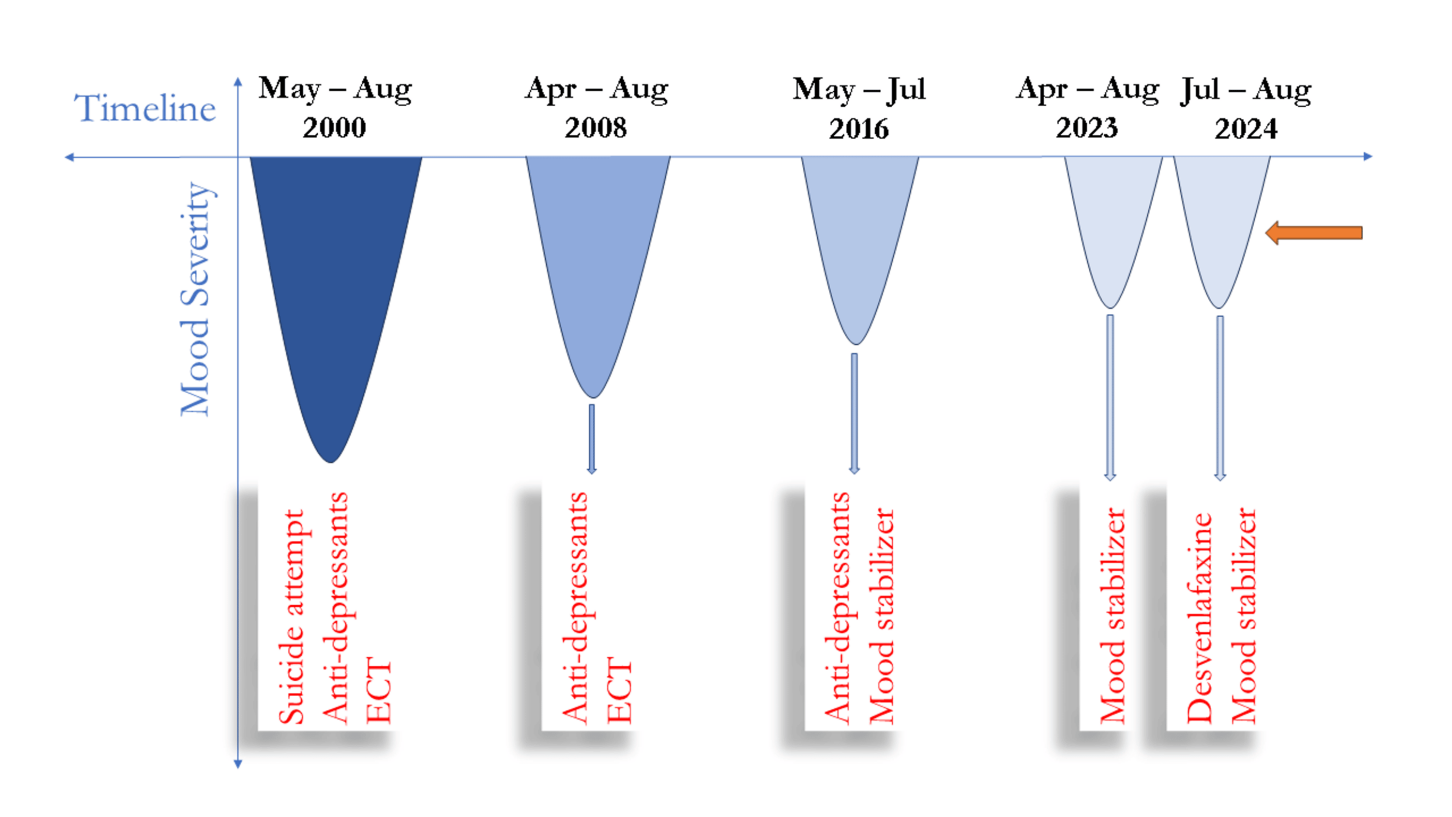
Clinical history across timeline ECT: electroconvulsive therapy

A 46-year-old male patient presented to the psychiatry outpatient department in July 2024 with a one-month history of depressive symptoms, including persistent low mood, anhedonia, fatigue, feelings of worthlessness, insomnia, and reduced appetite. He reported a significant decline in his interest in work, resulting in frequent absenteeism. Family members noted decreased communication and a preference for social isolation. On further exploration, the patient revealed a history of four prior depressive episodes, each occurring during the summer months. These episodes were marked by pronounced socio-occupational impairment, including withdrawal from social interactions and the necessity to leave his army deployment twice due to depressive symptoms. Following voluntary retirement, the patient also took a temporary break from his role as a lecturer during one such episode. The severity, duration, medication history, and treatment response for his depressive episodes are summarized in Figure 1.

During his first depressive episode, which occurred while he was serving in the army, the patient experienced symptoms of a similar nature, with severe disturbances in sleep and appetite. This episode was complicated by suicidal ideations and an alleged attempt at self-harm, where he tried to hang himself but was intercepted by a fellow officer. He was treated with multiple sessions of electroconvulsive therapy (ECT) alongside medications, resulting in remission of symptoms. ECT was also administered during his second episode in 2008. However, the exact details of the medications, their dosages, and the number of ECT sessions administered during these two episodes were unavailable.

In contrast, the subsequent three episodes were milder, with no reports of suicidal ideation. During the episodes in 2016 and 2023, the patient sought treatment from a local psychiatrist. Although information on the treatment plan from 2016 was unavailable, the patient recalled being informed about the addition of a mood stabilizer to his regular medication. Documentation from the 2023 episode revealed that the patient was prescribed oxcarbazepine at an initial dose of 150 mg twice daily, which was gradually titrated to 300 mg twice daily, leading to complete remission of symptoms.

Notably, none of these episodes correlated temporally with social, occupational, or financial stressors. All previous episodes resolved consistently with the onset of winter, during which the patient remained adherent to his prescribed medications. Following each episode, his medications were gradually tapered and ultimately discontinued over a period of six to nine months, as recommended by his psychiatrist; however, no documentary evidence of this tapering exists. Since the last episode, which occurred from April to August 2023, he had adhered to his prescribed medication of tablet oxcarbazepine 300 mg twice a day. The current depressive episode emerged while he was adherent to this treatment regimen.

The patient had no history of past or current psychiatric comorbidities, including substance use disorders, and no known medical or surgical illnesses. There was no significant family history of psychiatric conditions. He belongs to a middle-class family and resides with his wife and son. Currently, he is employed as a lecturer at a local engineering college.

Mental status examination revealed a depressed mood, restricted affect, and depressive thought content, with no evidence of suicidal ideation. His score on the Hamilton Rating Scale for Depression (HAM-D) was 18, indicating moderate severity of depression [7]. General physical examination findings, including pulse rate (72 beats per minute), blood pressure (116/68 mmHg), and body mass index (24.3), were normal. Systemic examination findings were also unremarkable. Routine laboratory investigations showed normal results, with hemoglobin 15.3 g/dL, white blood cell count 7,900/mm^3^, red blood cell count 5.08 million/mm^3^, platelet count 1.62 lakh/mm^3^, random blood glucose 79 mg/dL, blood urea nitrogen 12 mg/dL, and creatinine 0.9 mg/dL. Urine analysis was also within normal limits.

Given the severity of his symptoms, the degree of socio-occupational impairment, and the recurrent nature of his summertime depressive episodes, a diagnosis of major depressive disorder, moderate severity, with seasonal pattern was established in accordance with the DSM-5-TR criteria. The patient’s score on the Seasonal Pattern Assessment Questionnaire (SPAQ) was 19, which indicated a significant level of seasonality [8]. SPAQ has demonstrated utility in assessing seasonality across different geographic regions, including India [9].

The patient was started on desvenlafaxine at an initial dose of 50 mg once daily, which was increased to 100 mg once daily during a follow-up visit two weeks later. His ongoing mood stabilizer, oxcarbazepine, was continued at a dosage of 600 mg daily in two divided doses. This treatment regimen led to significant improvement, with remission achieved over four to six weeks. Currently, he is maintained on the same medications, with plans to continue this regimen for several months before considering a gradual tapering.

At his subsequent evaluation, his HAM-D score had reduced to seven, indicating the absence of depressive symptoms. Psychoeducation was provided, focusing on his diagnosis, prognosis, and early signs of relapse. Despite this, the patient opted not to engage in psychotherapy. He remains under regular follow-up, with his most recent visit in early November showing sustained improvement. His socio-occupational functioning has significantly enhanced, as evidenced by improved communication and consistent work performance.

## Discussion

Research indicates that summertime SAD appears less commonly than winter SAD and is often linked with features like irritability, insomnia, and weight loss, aligning it with non-seasonal depression symptoms, like in our case [10]. Studies conducted in high-latitude countries and regions with significant seasonal changes support an increased prevalence of summertime SAD near the equator, where summer durations are longer and more intense [11]. An Indian study found that patients with mood and somatic symptom disorders more frequently sought psychiatric care during hotter months [12]. Furthermore, another study analyzing language patterns on platforms like X (formerly Twitter) (X Corp., San Francisco, CA, US) found that high temperatures correlated with increased expressions of depressive symptoms, signifying seasonal influences on mental health expressions across large populations [13].

Numerous potential mechanisms have been theorized for this phenomenon. Elevated ambient temperatures stimulate the hypothalamic-pituitary-adrenal axis and can elevate cortisol and catecholamine levels, promoting stress responses and potential dysregulation in neurotransmitter levels essential for mood regulation [14]. Additionally, heat stress is believed to affect brain oxygenation and cooling, particularly under prolonged high temperatures. This effect can occur through vasodilation and fluid loss from dehydration, potentially impairing blood flow to emotional regulation centers such as the amygdala and prefrontal cortex [15]. This dysregulation may also contribute to mood instability, further supporting the pathophysiological model for summertime SAD. In regions with high pollen counts, immune responses triggered by allergens, resulting in cytokine and interleukin activation, have been associated with changes in neurotransmitter levels, potentially linking summertime SAD through inflammatory pathways [16]. Apart from environmental factors, socio-cultural influences like higher individualism have been correlated with winter SAD, while collectivist, lower-power-distance societies, often exposed to more sunlight, aligned with summer-type SAD manifestations [17]. These associations suggest that both environmental and cultural factors could have played their roles in the seasonal expression of mood disorder in our patient.

The potential mental health consequences of rising global temperatures, driven by climate change, are an area of growing research interest [18]. Tropical countries like India are particularly vulnerable to the effects of increasing heat, which may contribute to a higher incidence of summertime depression and other psychiatric conditions. This underscores the urgent need for comprehensive research aimed at understanding the underlying causes, developing effective treatment strategies, and identifying preventive measures to mitigate the impact of these environmental changes on mental health.

## Conclusions

Our case provides valuable insight into the clinical presentation of summertime SAD, a relatively rare and understudied condition, particularly within the Indian context. Recognizing the environmental, biological, and social factors that contribute to summertime depression is essential for accurate diagnosis and effective management. Further research is warranted to explore the prevalence, pathophysiology, and socio-cultural determinants of summertime SAD, especially in tropical and equatorial regions where climatic conditions may play a more significant role in the future.
